# New Insights into Mechanisms of Cardioprotection Mediated by Thyroid Hormones

**DOI:** 10.1155/2013/264387

**Published:** 2013-03-10

**Authors:** G. Nicolini, L. Pitto, C. Kusmic, S. Balzan, L. Sabatino, G. Iervasi, F. Forini

**Affiliations:** ^1^Institute of Clinical Physiology, CNR, 56100 Pisa, Italy; ^2^CNR, Tuscany Region G. Monasterio Foundation, Pisa, Italy

## Abstract

Heart failure represents the final common outcome in cardiovascular diseases. Despite significant therapeutic advances, morbidity and mortality of heart failure remain unacceptably high. Heart failure is preceded and sustained by a process of structural remodeling of the entire cardiac tissue architecture. Prevention or limitation of cardiac remodeling in the early stages of the process is a crucial step in order to ameliorate patient prognosis. Acquisition of novel pathophysiological mechanisms of cardiac remodeling is therefore required to develop more efficacious therapeutic strategies. Among all neuroendocrine systems, thyroid hormone seems to play a major homeostatic role in cardiovascular system. In these years, accumulating evidence shows that the “low triiodothyronine” syndrome is a strong prognostic, independent predictor of death in patients affected by both acute and chronic heart disease. In experimental models of cardiac hypertrophy or myocardial infarction, alterations in the thyroid hormone signaling, concerning cardiac mitochondrion, cardiac interstitium, and vasculature, have been suggested to be related to heart dysfunction. The aim of this brief paper is to highlight new developments in understanding the cardioprotective role of thyroid hormone in reverting regulatory networks involved in adverse cardiac remodeling. Furthermore, new recent advances on the role of specific miRNAs in thyroid hormone regulation at mitochondrion and interstitial level are also discussed.

## 1. Thyroid and Cardiac Dysfunction: A Not Yet Well-Understood Relationship

Cardiac performance is determined by the coordinated and dynamic interaction between several cell types and various components of the extracellular matrix (ECM). In response to stress conditions such as acute ischemia, pressure and volume overload and congenital and acquired valvular cardiac diseases, heart evokes a diverse and complex array of cellular responses involving both cardiomyocytes (CMs) and nonmuscle cells that initiate and sustain a process of structural remodeling of the myocardium. Cardiac remodeling (CR) is clinically manifested by changes in the size, shape, and function of the heart. From a histopathological point of view, it is characterized by structural derangements and rearrangements of the tissue components of the myocardial chamber wall and it involves CM death, cardiofibroblast (CF) proliferation, fibrotic processes, rarefaction of coronary vasculature, and CM hypertrophy [1]. Ultimately cardiac remodeling leads to heart failure (HF), a final threatening condition with poor prognosis. Prolonged treatment with angiotensin converting enzyme inhibitors and beta-adrenergic receptor antagonists (*β*-blockers) has partially attenuated the onset and the progression of the adverse process; nonetheless the associated morbidity and mortality remain unacceptably high. Undoubtedly, new and more efficacious interventions are highly desired to counteract the progression towards HF. Moreover, an inexpensive treatment targeting the fundamental causes of myocardial remodeling would be more appealing since this would combine an improved outcome with reduced health care costs. As in the case of aspirin and *β*-blockers, the application of existing drugs to new target of diseases and disorders may be a valid alternative to the development of new drugs and pharmaceutical formulas.

Over the past few years, a virtual explosion of new knowledge took place in many biomedical fields. Among these, a primary position is occupied by the physiological and pathophysiological relationship that exists between the thyroid gland and the cardiovascular (CV) system.

The influence of thyroid hormones (THs) on the CV system involves the regulation of key processes related to maintenance of cardiac contractility, electrophysiological functions and cardiac structure [2–4]. To exert its effects, the principal product of thyroid gland T4 must be converted into its biologically active form, T3, by type 1 and type 2 deiodinase enzymes in peripheral tissues. 

Current evidence shows that T3 levels significantly decline after myocardial infarction (MI) both in animal models and in patients due to the reduced conversion of T4 into T3 and the concomitant increased conversion of T4 into the inactive rT3 by the upregulation of type 3 deiodinase [5].

Although low T3 state has been long time considered a beneficial adaptive mechanism activated under stress conditions [6], several studies have shown that the low T3 syndrome may have an adverse prognostic impact on various acute and chronic cardiac disorders [7–11]. Importantly, many of the cardiac alterations observed in subclinical hypothyroidism are reversed once thyroid function has been normalized [12, 13]. 

On the basis of the evidence obtained from cells, animals, and even humans finding, it seems likely that timely treatments targeting the TH signaling may promote endogenous regeneration of the damaged myocardium [14–16]. Indeed, THs are critical modulators of myocardium development [17]. During the neonatal period, TH plasma levels increase promoting the switch from the fetal into the adult isoforms of key myocardial proteins such as myosin heavy chain (MHC) isoforms *α* and *β*. In the early phases of cardiac disease progression, the activation of a fetal gene reprogramming occurs as compensatory response that prompts cell de-differentiation [17]. In this context, low T3 level may favour cardiac repair but, in the long term, it can lead to end-organ damage. 

We will briefly review the major reported evidence supporting the hypothesis that a low thyroid signaling, namely, the one resulting in a postischemic myocardium is maladaptive (i.e., hypothyroid heart) and TH replacement, may counteract progression toward cardiac pathological remodeling and failure by inducing beneficial effects on myocyte survival, reduction of interstitial fibrosis in the non-infarcted myocardium, prevention of microvascular loss, and blunting pathological hypertrophy.

## 2. Thyroid Hormone Protective Action from Myocyte Death: The Intriguing Interplayer Role of Mitochondrion

CM death is now recognized as a critical factor in the development of heart diseases [18–22].

Myocyte apoptosis is an important event after acute myocardial infarction and may be responsible for a significant portion of myocyte death during the acute ischemic stage, as well as progressive loss of surviving myocytes during the subacute and chronic stages [23, 24]. In addition to the infarcted area, profound ongoing myocyte apoptosis has been demonstrated in the border area after MI [23, 24]. Moreover, inhibition of myocyte apoptosis improves left ventricular (LV) remodeling and cardiac function [25]. 

### 2.1. TH Activation of Intracellular Kinase Signaling

THs have been shown to limit cell apoptosis under stress conditions in several models. One of the major cardioprotective mechanisms involves the activation of phosphatidylinositol 3′ kinase/protein kinase B (PI3K/Akt) and extracellular regulated kinase 1 and 2 (ERK1/2) signaling. T3 replacement in CM cultures prevented serum starvation-induced apoptosis through an Akt-mediated mechanism [14]. Accordingly, T3 can inhibit CM apoptosis and activate Akt signaling in the border area after acute myocardial infarction [26]. In a Langendorff rat heart model of ischemia reperfusion injury, T3 treatment during the reperfusion period significantly enhanced the recovery of function and reduced myocyte apoptosis through the activation of the PI3K/Akt and ERK1/2 axis [27].

### 2.2. Mitochondrial-Mediated Prosurvival Effects

An emerging mechanism of cardioprotection implies regulation of mitochondria function and biogenesis. Actually, besides their contribution to bioenergetic function, mitochondria are integrally involved in regulating myocardial calcium flux, myocyte cell death and remodeling events, reactive oxygen species (ROS) generation and antioxidant response, and in furnishing cardioprotective responses to physiological insults [28]. Not surprisingly, mitochondrial dysfunction plays a critical role in the occurrence and progression of HF [28, 29] underlining the importance of new therapeutic strategies aiming to preserve mitochondrial integrity.

THs are well-known regulators of mitochondrial biogenesis and function [30, 31]. Changes in thyroid status are associated with bioenergetic remodeling of cardiac mitochondria and profound alterations in the biochemistry of cardiac muscle, with repercussions on its structure and contractility [32]. A recent study reported that early T3 administration prevents cardiac remodeling and decreases death of stressed CMs by rescuing mitochondria function and biogenesis in a model of postischemic HF. The proposed mechanisms underlying the T3 beneficial effects are the upregulation of mitochondrial transcription factor A (mtTFA) and peroxisome proliferator activated receptor gamma coactivator 1 alpha (PGC-1*α*) and the opening of the protective mitochondrial ATP-dependent potassium channel (mitoK-ATP) [22]. 

Recently, it has been demonstrated that HF can also be aggravated through myocyte necrosis which is largely initiated at the level of the mitochondria following Ca^2+^ overload [21]. Aberrant Ca^2+^ homeostasis is a universal feature of human and experimental HF and is widely attributed to decreased sarcoplasmic reticulum calcium ATPase (SERCA) expression or chronic SERCA inhibition by phospholamban (PLB) [33]. Reduction of SERCA level in cardiac disease enhances cytosolic Ca^2+^ overload which besides compromising CM contractility, initiates a pathway cascade leading to mitochondrial dysfunction and necrotic cell death. 

THs are well-established potent regulators of SERCA2a pump expression and cardiac muscle contractility [34]. Interestingly, there is a very close correlation between the rate of sarcoplasmic reticulum Ca^2+^ uptake and the PLB to SERCA2a ratio in hypothyroid, euthyroid, and hyperthyroid hearts, and this determines the contractile effectiveness of the heart [35].

Evidences has provided that the diminished expression of the SERCA associated to hypothyroidism can be restored by correcting the hormonal disorder through TH therapy [36, 37]. Moreover, in an ex vivo study on human myocardial biopsies, T3 supplementation at physiological doses restored Ca^2+^ handling which was altered after long-term T3 deprivation [38]. Thus, it is likely that the low-T3 state, observed during the evolution of cardiac disease, may favor adverse remodeling by inducing a reduction of mitochondrial biogenesis and function and by enhancing the activation of necrotic pathways driven by mitochondrial Ca^2+^ overload.

### 2.3. Role of miRNA Regulation in Survival Process

Recently, some miRNAs have been identified with critical importance in inhibiting mitochondrial-mediated cell death pathways [39, 40]. In particular miRNA-30 family members are abundantly expressed in the heart, but their levels decreased in response to CM oxidative stress or myocardial ischemia/reperfusion [41–43]. Preliminary, unpublished data from our laboratory strongly indicate that in a rat model of myocardial ischemia/reperfusion, early three-day infusion of physiological doses of T3 is able to maintain myocardial contractility and mitochondrial function in the border area of the ischemic injury and that this effect is linked to the maintenance of miRNA-30a expression levels.

In summary, overall available data support the hypothesis that an antiapoptotic and antinecrotic effect of TH may be important in preventing adverse cellular responses in cardiac disease and that this effect seems to be mediated, at least in part, by a TH action on cardiac mitochondrion.

## 3. Thyroid Hormone Homeostatic Action on Cardiac Interstitium and Vasculature 

In addition to CMs, the heart contains many other “non-CM” cell types, such as fibroblasts, endothelial cells, smooth muscle cells, and immune cells, which play a fundamental role in both physiological and pathological condition. 

### 3.1. TH Effect on Cardiac Fibrosis/Interstitium

CFs are responsible for maintaining the structural integrity of the heart. As reported by many investigators, in the clinical context of myocardial damage, CF undergoes a transformation from a quiescent cell, primarily involved in the extracellular matrix homeostasis, to myoCF, an activated cell type which plays a central and dynamic role in wound healing. The process of cellular metamorphosis is associated with several different phenotypic endpoints including cellular proliferation, chemotactic migration, ECM remodeling and the coordinated production of specific cytokines, interleukin (e.g., IL-1 and IL-6), chemokine, and growth factors (e.g., TNF-*α* and TGF-*β*) [44–50]. Among the main effects of ECM remodeling, the continuous synthesis and deposition of interstitial collagen, along with deregulation of matrix metalloproteinases (MMPs) and tissue inhibitors of MMPs (TIMPs), impair the cardiac diastolic function and compromises the systolic mechanics leading to HF progression [47, 51–54].

THs seem to play a pivotal role in ECM homeostasis. However, the only available data deal with hyperthyroidism or hypothyroidism experimental models. It has been reported that mRNA for collagen type I, the major fibrillar collagen in the heart, is downregulated by TH treatment both in the myocardium and in CF culture [55]. In similar experiments TH-induced hypertrophy is accompanied by an increase of MMP1, MMP2, and TIMP2 without signs of cardiac fibrosis or inflammatory response [56, 57]. T3 can also repress the activation of some transcription factors such as activator protein 1 (AP-1) [58, 59] that are involved in activation of MMPs and collagen gene expression [60]. 

Hypothyroidism condition increased expression of collagen precursor in rat CF culture, while TH treatment reversed this effect only in the presence of thyroid hormone receptor beta and alpha (TR*β* and TR*α*) [59].

Altogether, the above findings suggest that TH may selectively blunt the onset of cardiac fibrosis and play an important role in regression of cardiac fibrosis via endocrine pathways [55]. However, the effect of physiological TH treatment on ECM remodeling in oxidative stressed myocardium has never been reported. We believe that unravelling this important issue with further translational studies may be crucial in the perspective of a new therapeutic approach with TH in cardiac disease.

### 3.2. Role of miRNAs in Cardiac Fibrosis

Recent studies have shown that the expression of several miRNAs which play direct and indirect roles in the regulation of cardiac fibrosis is altered following MI or other fibrotic pathologies [41, 61]. Among them miRNA-29 family plays an important antifibrotic role and is downregulated in models of cardiac ischemia and hypertrophy [43, 61].

The miRNA-29 family comprises three members miRNA-29a, 29b, and 29c, which are all predominantly expressed in fibroblast within the heart [61]. Knockdown of miRNA-29 resulted in the upregulation of collagens in the heart, suggesting that miR 29 indeed acts an inhibitor of cardiac fibrosis [61]. Unpublished data from our laboratory indicate that early T3 replacement after ischemia/reperfusion in rat induces the expression of miRNA-29c which is associated to the maintenance of cardiac performance. Globally, these results suggest that the downregulation of miRNA-29 contributes to cardiac fibrosis and that strategies to maintain miRNA-29 expression may be beneficial in the setting of fibrotic disease.

### 3.3. TH Effects on Coronary Vasculature

Besides CMs and CFs, both endothelial cells and microvasculature play a key role in regulating and maintaining cardiac function. Insufficient angiogenesis is one of the causes of myocardial dysfunction and there is strong evidence of reduced myocardial capillary density in several human heart disease such as aortic stenosis, dilated cardiomyopathy, and ischemic cardiomyopathy [62]. 

TH has well-documented effects on angiogenesis [63–65]. TH-induced sprouting of endothelial cells has been documented in different models such as the chick chorioallantoic membrane and cultured left ventricle tissue [66, 67]. The molecular mechanism of the proangiogenic action of TH is initiated nongenomically at plasma membrane of endothelial cells (ECs) through the interaction with integrin *α*v*β*3. The transduction of the hormone signal is mediated by mitogen-activated protein kinase ERK1/2 and results in downstream transcription of several angiogenesis-relevant genes such as basic fibroblast growth factor (bFGF) and vascular endothelial growth factor (VEGF) [68]. This pro-angiogenic effect may be potentiated by a crosstalk between the integrin receptor for TH and the VEGF and bFGF receptors [69]. Iodothyronines seem also to stimulate angiogenesis through enhancing the expression of hypoxia inducible factor 1 alpha (HIF-1*α*), a transcription factor important for ischemia-induced coronary artery collateralization [22, 70]. The proposed mechanism involves the interaction of the hormone with TR*β* in the cytosolic compartment and the activation of PI3K signaling [71, 72]. 

The contractile component of blood vessel, the vascular smooth muscle cell, is also influenced by TH action. In a previous study, we reported that T3-induced arteriolar dilation in a nongenomic way, likely enhancing nitric oxide production. In this model, the local conversion of T4 to T3 appears to be crucial for the dilation induced by T4 administration [73]. Recently, TH has been shown to induce coronary relaxation by a TR*α*-dependent mechanism. TR*α*-knockout (TR*α*-KO) mice exhibited increased coronary contraction. “In vitro” TH treatment of coronary smooth muscle cell isolated from TR*α*-KO mice decreased contractility by enhancing K^+^ channel activity [74]. Moreover, T3 administration suppressed cytokine expression and vascular smooth muscle cell proliferation in an experimental model of coronary atherosclerosis. This effect was, at least in part, dependent on the inhibition of angiotensin II signalling [75]. 

The important role of TH in the regulation of cardiac vasculature is further confirmed by the impaired coronary artery contraction, the reduced density of small arterioles, and the altered echocardiographic parameters reported in low thyroid function [76]. 

## 4. TH Antagonizes Pathological Hypertrophy 

CR observed during progression of cardiac disease includes concentric and eccentric hypertrophy of surviving CMs.

The pathological hypertrophy of the human heart has been described as the single most important independent risk factor for increased mortality [77, 78]. Cardiac hypertrophy develops as an overall adaptive response to stress conditions; signaling pathways activated in CMs and non-CMs by various noxious stimuli (e.g., neurohormonal stimulation and oxidative stress) should be tightly regulated to maintain cardiac homeostasis and prevent pathological cardiac hypertrophy. Maladaptive hypertrophy is associated with impaired myocardial capillary growth, unfavourable changes in ECM composition and fibrosis. It is sustained by elevation of angiotensin II, TNF-*α*, and catecholamine plasma levels and seems to involve downstream pathways such as c-Jun N-terminal kinase and p38 [79, 80].

On the contrary, physiological hypertrophy is characterized by parallel growth of vascular network, absence of fibrosis, and lack of fetal gene reprogramming [81]. TH enhances many of the signaling pathways activated during cardiac physiological growth including PI3K/AKT/mTOR and GSK3*β* axes [82**]. **THs have been recognized to induce physiological growth of the heart through genomic regulation of specific target genes that encode both structural and functional proteins [82]. In CMs, TH upregulates SERCA2, *α*-MHC, Na/K-ATPase, and K^+^ channel while downregulates *β*-MHC, PLB, TR*α*1, and Na/Ca exchanger [83]. Thus, THs regulate expression of specific genes of critical importance for cardiac contractility and prevent the expression of the fetal gene program characteristic of pathological hypertrophy [2, 84].

### 4.1. Regulation of MHC Gene Switching

The mechanisms that regulate MHC gene switching by TH have been the focus of intense interest. Besides to genomic action, TH can affect myocardial MHC composition through epigenetic modification and miRNA regulation. In hypothyroid rat, treatment with trichostatin A, an inhibitor of histone deacetylation, together with T3 significantly increased the transcription of *α*-MHC, thus demonstrating a potential role for histones as cofactors in the T3 regulation of cardiac *α*-MHC transcription [85]. 

Recently, a family of miRNAs (miRNA-208a, miRNA-208b, and miRNA-499), localized within the MHC genes loci, has been shown to form an intricate regulatory network together with their host genes to regulate cardiac hypertrophy [86, 87]. These miRNAs are coexpressed and are regulated together with their host MHC genes by the same transcriptional events and signaling pathways. miR-208a is a cardiac-specific miRNA encoded by the *α*-MHC gene. While physiological levels of miR-208a are required for proper cardiac electrical conduction, a fourfold increase of miR-208a induces hypertrophic growth in mice heart by suppressing thyroid hormone-associated protein 1 and myostatin, which are negative regulators of muscle growth and hypertrophy [88]. These results clearly demonstrate the pathological consequences deriving from the perturbed expression of a single miRNA. Deregulation of TH signaling in cardiac disease leads to *α*-MHC/miRNA-208a inhibition while in vitro treatment with THs significantly upregulates *α*-MHC/miRNA-208a expression and reduces *β*-MHC/miRNA-208b expression [88]. miRNA-208b and miRNA-499 play redundant role in *β*-MHC upregulation and seems to be regulated in the same way by TH [87]. This data suggests that physiological TH concentrations are required to ensure proper miRNA levels and to preserve MHC composition.

The observation that TH activates many of the beneficial aspects of physiological hypertrophy has raised the possibility of their therapeutic utility in the treatment of the post-infarcted heart or in HF [4, 89, 90].

## 5. Conclusive Remarks

Experimental and clinical studies strongly support the concept that TH plays a fundamental role in cardiovascular homeostasis in both physiological and pathological conditions.

Increasing evidence indicates that the low TH is not simply a consequence of cardiac disease evolution but a permissive state that favours adverse cardiac remodeling and failure.

**Figure 1:**
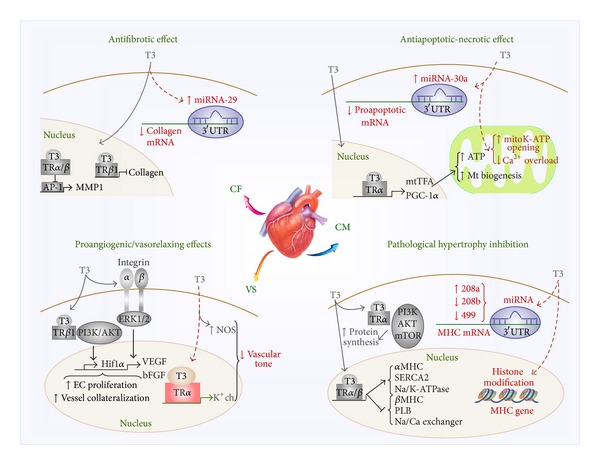
Schematic representation of cardioprotective mechanisms of T3. Coloured details refer to emerging findings, and established mechanisms are depicted in gray scale. Top left: antifibrotic effect on CF. T3 binding to TR*β*/*α* represses AP-1 activity resulting in MMP1 downregulation. Moreover T3 inhibits collagen synthesis through the classical genomic pathway mediated by TR*β*1. The upregulation of the antifibrotic miRNA-29 by T3 opens a new window of investigation. Bottom left: proangiogenic and vasorelaxing effects of T3 on VS. T3 stimulates EC proliferation through the interaction with a plasma membrane integrin receptor. Signal transduction is mediated by activation of ERK1/2 and results in downstream transcription of proangiogenic genes such as VEGF and bFGF. The interaction of T3 with cytoplasmic TR*β*1 activates PI3K/AKT. The signal cascade leads to stimulation of Hif1-*α* with consequent increase of EC proliferation and vessel collateraization. In smooth muscle cells T3-mediated reduction of vascular tone is achieved by stimulation NOS activity and by the recently reported upregulation of K^+^ch. Top right: antiapoptotic and antinecrotic effects on CM. Classical genomic action of T3 increases PGC1-*α* expression which in turn up-regulates mtTFA; the resulting increase of mitochondrial biogenesis and function improves cell viability. As recently reported, T3 might limit mitochondrial-mediated apoptosis and necrosis by reducing mitochondrial matrix calcium overload and by favouring the opening of the cardioprotective mitoK-ATP channel. Upregulation of the antiapoptotic miRNA-30a pathway by T3 represents an emerging finding that encourages future researches. Right bottom: inhibition of CM pathological hypertrophy. Through the classical genomic mechanism, T3 regulates the expression of several genes critically involved in contractile function such as *α*/*β*-MHC, SERCA2, Na/K-ATPase, PLB, and Na/Ca exchanger. Another way of T3-mediated upregulation of protein expression requires the activation of the PI3K/AKT/mTOR axes. Histone modifications of MHC gene have been involved in T3-mediated regulation of MHC isoform composition. T3 can also affect myocardial MHC expression through regulation of a family of miRNAs (miRNA-208a, miRNA-208b, and miRNA-499) encoded by MHC genes.

As summarized in Figure 1, restoration of a normal TH profile might counteract the progression of adverse cardiac remodeling by (1) inhibiting CM death pathways, (2) reducing fibrous tissue deposition, (3) improving myocardial perfusion, and (4) preventing pathological cardiac hypertrophy. Regulation of miRNA expression and prevention of mitochondria-driven death pathways are emerging mechanisms of T3-mediated cardioprotection that deserve further investigation.

## Abbreviations

*α*-MHC: Alpha-myosin heavy chain
AP-1: Activator protein 1
*β*-blockers: Beta-adrenergic receptor antagonists
*β*-MHC: Beta-myosin heavy chain
bFGF: Basic fibroblast growth factor
CF: Cardiofibroblast
CM: Cardiomyocyte
CR: Cardiac remodeling
CV: Cardiovascular
EC: Endothelial cell
ECM: Extracellular matrix
ERK1/2: Extracellular regulated kinase 1 and 2
GSK3*β*: Glycogen synthase kinase 3 beta
HF: Heart failure
Hif1-*α*: Hypoxia inducible factor 1 alpha
IL-1: Interleukin 1
IL-6: Interleukin 6
K^+^ch: Potassium channel
KO: Knockout
LV: Left ventricular
miRNA: Micro RNA
mitoK-ATP: Mitochondrial ATP-dependent potassium channel
MMP: Matrix metalloproteinase
mTOR: Mammalian target of rapamycin
mtTFA: Mitochondrial transcription factor A
MI: Myocardial infarction
Na/Ca exchanger: Sodium potassium exchange channel
Na/K-ATPase: Sodium potassium ATPase
NOS: Nitric oxide synthase
PGC-1*α*: Peroxisome proliferator activated receptor gamma coactivator 1 alpha
PI3K/AKT: Phosphatidylinositol 3′ kinase/protein kinase B
PLB: Phospholamban
ROS: Reactive oxygen species
SERCA2: Sarcoplasmic reticulum calcium ATPase
rT3: Reverse T3
TH: Thyroid hormone
TIMPs: Tissue inhibitors of MMPs
TGF-*β*: Transforming growth factor beta
T4: Thyroxine
T3: 3,5,3-Triiodo-L-thyronine
TR*α*: Thyroid hormone receptor alpha
TR*β*: Thyroid hormone receptor beta
TNF-*α*: Tumor necrosis factor alpha
VEGF: Vascular endothelial growth factor
VS: Vasculature.
